# Uterine adhesion: Is luteal phase prior to follicular phase in uterine adhesiolysis?

**DOI:** 10.1097/MD.0000000000027194

**Published:** 2021-09-17

**Authors:** Yilin Fei, Jie Wen, Xingmiao Li, Ning Wang, Mo Chen, Xiuxiu Jiang

**Affiliations:** Women's Hospital, School of Medicine, Zhejiang University, Hangzhou City, Zhejiang Province, China.

**Keywords:** adhesiolysis, endometrial thickness, hysteroscopy, pregnancy rate, re-adhesion

## Abstract

To compare the patients’ outcomes of Asherman syndrome who underwent uterine adhesiolysis in luteal phase or follicular phase.

A retrospective cohort study.

A tertiary hospital in China.

Four hundred sixty-four women suffered intrauterine adhesion who underwent monopolar adhesiolysis from March 2014 to March 2017 were analyzed. One hundred seventy-eight patients underwent operations in follicular phase (OFP) and 286 underwent operations in luteal phase (OLP).

Hormone therapy was accompanied with an intrauterine device and a second-look hysteroscopy was performed postoperatively.

Endometrial thickness in women was analyzed by a transvaginal 3-dimensional ultrasound examination. Re-adhesion was confirmed by a second-look hysteroscopy 3 months after hysteroscopic adhesiolysis. Pregnancy rate was acquired by questionnaires 3 months after a second-look hysteroscopy.

OLP has advantages with thicker luteal endometrium (*P* = .001), higher pregnancy rates (*P* < .001), and lower re-adhesion rates (*P* = 0015) compared to these values of OFP.

For Asherman syndrome, our study showed that OLP is more feasible than OFP in intrauterine adhesiolysis.

## 1 Introduction

Intrauterine adhesion also known as Asherman syndrome, is caused by the trauma of pregnancy or non-pregnancy uterus, resulting in damage to the endometrial basal layer, partially or completely occlusion of the uterine cavity, manifesting as a series of syndromes, including secondary amenorrhea, pain, and infertility.^[1]^ In China, with the establishment of the 2-child policy, there are also a growing number of women holding out hope to get pregnant at risk of adhesiolysis. Hysteroscopy is the gold standard for diagnosing the disease.^[2,3]^ Hysteroscopic adhesiolysis is a routine procedure for the treatment of the disease, but pregnancy chances get less with the recurrence of the adhesion.^[4,5]^ Chinese intrauterine adhesion classification and scoring standards refer to the scoring scale proposed by the American Society of Fertility and the European Society of Gynecological Endoscopy, combined with the treatment effect and influencing factors of intrauterine adhesion, and included clinical indicators closely related to the treatment outcome. In this study, Chinese intrauterine adhesion classification and scoring standards were adopted as showed in Supplemental Digital Content.

In the course of hysteroscopic operation, adhesiolysis held the risk of causing endometrial injury. To reduce endometrial injury, method such as the separation of adhesions by scissor was recommended, nevertheless it has little effect on preventing relapse of adhesion.

There was no report regarding the operation timing on the prognosis of intrauterine adhesiolysis. To compare the effects of uterine adhesiolysis operated in luteal phase and follicular phase, we performed a retrospective study to analyze the endometrial thickness, pregnancy rate, and re-adhesion rate postoperatively.

## 2 Materials and methods

Our study is registered under the Chinese Clinical Trial Registry (No: chictr-irc-17013426). This study was approved by the Ethics Committee Board of the Women's Hospital, School of Medicine, Zhejiang University (No: 20170130), and informed consent was obtained from the patients.

A retrospective analysis was conducted from March 2014 to March 2017 at the Women's Hospital, School of Medicine, Zhejiang University. The inclusion criteria were followed by: patients with moderate and severe uterine adhesion were diagnosed by 2 qualified surgeons with class III and above through hysteroscopy according to the Chinese intrauterine adhesion classification and scoring standards (shown in Supplemental Digital Content); patients were aged 20 to 40 years old; the intrauterine device (IUD) all the same type was placed immediately and hormone therapy was prescribed for 3 months after operation; a second-look hysteroscopy was performed 3 months after operation; serum levels of reproductive hormones were in fertility level. The exclusion criteria were followed by: genital tract diseases associated with infertility including hydrosalpinx, endometriosis, and pelvic tuberculosis; abnormal liver function; intra-operative or postoperative complications, such as uterine perforation, hyponatremia, cerebral edema, hemorrhage, and pelvic infection; taking medicine including traditional Chinese medicine; Assisted Reproductive Technology such as intrauterine insemination, in vitro fertilization-embryo transfer was used after operation; spouses of infertility. Initially, a total of 559 patients were recruited, meanwhile, 74 of them were lost to follow-up, and 21 patients were excluded due to the refusal to a second-look hysteroscopy. Finally, 464 patients were included in the study.

According to the operation timing, 178 patients were divided into the operations in follicular phase (OFP) and 286 patients were divided into the operations in luteal phase (OLP). The surgeries were performed by 2 qualified surgeons with class III and above. Epidural anesthesia was used, and the operation method was 100 to 240 V monopolar hysteroscopic adhesiolysis (monopolar electrocoagulation is routinely used in this hospital, and there has never been an electrical conduction accident, so bipolar electrocoagulation is not used). The operations were completed within 30 minutes, and the volume of fluid distension medium used was less than 3000 mL. The IUD all the same type was immediately placed for 3 months and taken away by the second-look hysteroscopy which was performed by the same 2 qualified surgeons with class III and above to observe the recurrence of the adhesions 3 months after adhesiolysis. The hormone therapy was prescribed for 3 months postoperatively to promote the repair of the endometrium. A daily oral dosage of estradiol valerate (Progynova, Bayer Medical Care Co. Ltd.) was 2 mg tid for 21 days, and oral progestogen (Duphaston, Abbott) was prescribed at 10 mg bid during the last 10 days of the cycle. The investigation included double-layered endometrial thickness, re-adhesion rate, and pregnancy rate.

### 2.1 Statistics

Statistical analysis was performed with SPSS16.0 software (SPSS Inc., Chicago, IL). Continuous data were expressed as mean ± standard deviation or median. Categorical data were presented as frequencies. Differences between the 2 groups were analyzed with the Chi-squared test and *t* test or rank sum test. *P* < .05 was considered statistically significant.

## 3 Results

Four hundred sixty-four patients underwent uterine adhesiolysis in which 178 (38%) patients were included in OFP, and 286 (62%) patients were included in OLP. There were no significant differences in age (range, 20–40 years old) which were divided into 2 groups: less than or equal to 35 years old and older than 35 years old (*P* = .167). Previous history of uterine cavity surgery was divided into 2 groups: less than or equal to 3 times and more than 3 times. Previous abortion history was divided into 2 groups: less than or equal to 3 times and more than 3 times. Thus, the 2 groups which had no significant differences in age (*P* = .167), previous history of uterine cavity surgery (*P* = .156), and previous abortion history (*P* = .195) were comparable as shown in Table 1.

**Table 1:** Outcomes of uterine adhesion in different operation timings.

	OFP	OLP	*P*
No. of patients	178	286	–
Age > 35 yrs	50	64	.167
Age ≤ 35 yrs	128	222	
History of previous operations > 3	6	3	.156
History of previous operations ≤ 3	172	283	
History of abortions > 3	35	43	.195
History of abortions ≤ 3	143	243	
Endometrial thickness (mm) before operation	4.6	5.0	.498
Endometrial thickness (mm) after operation	5.0	5.5	.001
Pregnancy rate (%)	20.8	41.3	<.001
Re-adhesion rate (%)	31.5	21.3	.015

OFP = operations in follicular phase, OLP = operations in luteal phase.

In our study, the median thickness of the double-layered endometria in the luteal phase before operation in OFP was 4.6 mm and the median thickness of the double-layered endometria in the luteal phase before operation in OLP was 5.0 mm. The same intervention was performed between the 2 groups postoperatively. The IUD was immediately placed after adhesiolysis for 3 months and taken away by the second-look hysteroscopy. The hormone therapy was prescribed for 3 months postoperatively. Three months after the adhesiolysis, a transvaginal 3-dimensional ultrasound examination was performed to record the thickness of the double-layered endometria. The median thickness of the double-layered endometria in the luteal phase after operation in OFP was 5.0 mm while the median thickness of the double-layered endometria in the luteal phase after operation in OLP was 5.5 mm. There was no significant difference in endometrial thickness before operation in OFP and OLP (*P* = .498), whereas we found that the double-layered endometrial thickness after operation varied in different operational timing. The thickness of the endometria after operation in the OLP was higher than that in the OFP (*P* = .001); therefore, the difference between the 2 groups was significant. The comparison of the thickness of the double-layered endometria in the luteal phase of the 2 groups is shown in Table 1.

The comparison of the re-adhesion rate between the 2 groups was studied 3 months after hysteroscopic adhesiolysis. After the operation, every patient was treated immediately with an IUD and the hormone therapy for 3 months. The second-look hysteroscopy was performed by the same 2 qualified surgeons with class III and above to observe the recurrence of the adhesions. The recurrence rate of the uterine adhesions 3 months after operation in the OFP was 31.5% (56/178) and 21.3% (61/286) in the OLP. In short, the recurrence rate was significantly different in different operation timing (*P* = .015) and was shown in Table 1.

On the other hand, a comparison of the pregnancy rate between OFP and OLP was done 3 months after the second-look hysteroscopy. After hysteroscopic adhesiolysis, every patient was immediately treated with the same type of IUD and hormone therapy for 3 months. Then the second-look hysteroscopy was performed.^[6]^ The follow-up questionnaires were carried out 3 months after the second-look hysteroscopy. Pregnancy confirmed by ultrasound was categorized as pregnancy, whereas infertility, miscarriage, and ectopic pregnancy confirmed by ultrasound were not categorized as pregnancy through questionnaires. The postoperative pregnancy rate between OFP and OLP was compared. The results showed that the pregnancy rate was 41.3% (118/286) in OLP and 20.8% (37/178) in OFP. This outcome demonstrated that operation timing (OFP and OLP) on the rate of pregnancy was significantly different (*P* < .001), and the pregnancy rate in OLP was higher than that in OFP. The results were shown in Table 1.

## 4 Discussion

At present, there have been studies performed on the comparison between the effects of hysteroscopic adhesiolysis and separation by scissors.^[7,8]^ The advantages of scissors separation are to avoid the electrothermal effect and damage of energy instruments on normal endometrium around scars, reduce wound exudation, and reduce the formation of postoperative re-adhesion. However, due to the special anatomical morphology of the uterine cavity and the type of adhesion, the use of these devices may be restricted, especially for the separation of muscular peripheral adhesions, which is not only difficult to operate, but also difficult to stop bleeding on the wound. This method is not suitable for moderate to severe intrauterine adhesion operation. Hysteroscopic adhesiolysis is an indispensable choice for the treatment of moderate to severe intrauterine adhesion, especially for the separation of peripheral muscular adhesions, because it is simple and effective to separate and resect the adherent scar tissues by using energy-interventional electrodes. But there is no study regarding the comparison of the prognosis of different timings of uterine adhesiolysis. Based on our study, we compared the double-layered endometrial thickness, pregnancy, and re-adhesion rates of OFP and OLP.^[9]^ OLP has advantages with thicker luteal endometria (*P* = .001), higher pregnancy rates (*P* < .001), and lower re-adhesion rates (*P* = .015) compared to these values of OFP.

The postoperative regeneration of the endometrium depends on 2 conditions: the destruction of the original base layer and the regeneration of glandular epithelial cells. If adhesiolysis causes damage to the endometrial base layer or even the endometrial stem cell layer, the regeneration of the endometrium may be very difficult, and this condition may also limit the growth of the endometrium.^[10,11]^ The endometrium in luteal phase is relatively thick and it is not easy to damage the functional layer during operation. Therefore, the operation during the luteal stage has a protective effect on the endometrium. Hormone therapy is used to promote the repair of the endometrium, but the basis of the drug depends on the amount of estrogen receptors in the endometrium.^[12,13]^ If there is less damage to the endometrium, more estrogen receptors are retained, and thus, the drug effect would be more responsive. Estrogen can effectively promote the thickening of the endometrial basal layer^[14,15]^ and the proliferation of glands, interstitial tissues, and blood vessels.^[16,17]^ Different operation timings have different effects on the pregnancy rates. In our study the pregnancy rate in OLP was higher than that in OFP. Pregnancy is a complex event, the regeneration and repair of the endometrium are crucial in pregnancy.^[18]^ Such as the early embryo develops into a structure called the blastocyst, which implants in the lining of the uterus. Implantation triggers the development of the placenta from fetal membranes. During this time, the fetus is nourished in 2 distinct ways – first, by glands in the uterus that feed into the intravillous space of the placenta, and later, by the maternal blood, which passes directly to the developing placenta.^[19]^ Patients with intrauterine adhesions tend to have lower pregnancy rates due to a poorly repaired endometrium. Intrauterine adhesion may lead to difficulty in endometrial regeneration because of the decrease of endometrial glands. Adhesiolysis, which further aggravates the injury of the endometrial layer, will make the regeneration of the endometrium even harder after an operation. The latest research has found that the distribution of blood vessels and blood perfusion changed after the injury to the endometrium and myometrium in patients with intrauterine adhesions. This change may lead to a decrease in the endometrium receptivity.^[16]^ As a result, the implantation of the embryo and the development of the placenta may be severely affected, even leading to cause infertility or abortion. Thickening of the endometrium is suitable for the implantation of embryos and further improves the clinical pregnancy rate. Based on the results obtained, the pregnancy rate after the operation of the luteal phase is higher than that of the follicular phase. Therefore, we suggest that the luteal phase is preferred for uterine adhesiolysis, as the endometrium has certain thickness in comparison to the follicular phase.^[2]^ Due to sufficient thickness of the endometrium, the damage by the electric current on the base layer may be less than that in the follicular operation. This effect will be beneficial to the recovery of the endometrium after operation, and in the long run, it helps with pregnancy.

Different operational timings have different effects on the recurrence of adhesion. If the electric current damages the base layer, the effect is irreversible. However, the endometrium base layer is more vulnerable to damage during the follicular phase. The inflammatory factors, fibrous tissue proliferation, formation and degradation of extracellular matrix, and the inflammatory reaction of the endometrium may inhibit the regeneration of the endometrium and promote the fibrotic growth, leading to the formation of fibrotic connective tissue.^[20]^ Exposing the intermuscular blood vessels and myometrium to the uterine cavity can cause the formation of adhesions between the anterior and posterior walls of the uterus. Therefore, we suggest that the operation should be performed when the endometrial layer is thickened to minimize the damage to the base layer. The recurrence of intrauterine adhesions is a nodus that surgeons often encounter. Therefore, adhesiolysis by hysteroscopy under direct vision is recommended as the treatment for symptomatic intrauterine adhesions.^[21]^ The endometrium is rather thin right after menstruation. Hence, OFP is traditionally considered to be the best time for adhesiolysis. As far as we are concerned, there is no study that has depicted the effect of different surgical timings on the recurrence and healing of adhesions under hysteroscopic adhesiolysis. Our findings suggest that the recurrence of postoperative adhesions in the OLP is less than that in the OFP. These results demonstrate that OLP may help in reducing the damage to the basal layer and in preventing the recurrence of adhesions.

## Author contributions

**Conceptualization:** Xiuxiu Jiang.

**Data curation:** Jie Wen.

**Formal analysis:** Yilin Fei.

**Investigation:** Yilin Fei, Mo Chen.

**Methodology:** Yilin Fei.

**Resources:** Yilin Fei, Xingmiao Li, Ning Wang.

**Writing – original draft:** Yilin Fei.

**Writing – review & editing:** Xiuxiu Jiang.

## Supplementary Material

SUPPLEMENTARY MATERIAL
