# Supplementary material for: Uterine adhesion: Is luteal phase prior to follicular phase in uterine adhesiolysis?
Source: Medicine (Baltimore). 2021 Sep 17;100(37):e27194. doi: 10.1097/MD.0000000000027194 (PMC8448072; doi:10.1097/MD.0000000000027194)
Supplement: SUPPLEMENTARY MATERIAL [file medi-100-e27194-s001.doc]

Chinese IUA classification and scoring standards

| [evaluation](../../../../C:/Program%20Files%20(x86)/Youdao/Dict/8.3.1.0/resultui/html/index.html#/javascript:;) [item](../../../../C:/Program%20Files%20(x86)/Youdao/Dict/8.3.1.0/resultui/html/index.html#/javascript:;) | project standard description | score |
| --- | --- | --- |
| scope of adhesion | <1/3 | 1 |
|  | 1/3~2/3 | 2 |
|  | >2/3 | 4 |
| adhesion properties | membranous | 1 |
|  | [fibroid](../../../../C:/Program%20Files%20(x86)/Youdao/Dict/8.3.1.0/resultui/html/index.html#/javascript:;) | 2 |
|  | muscular | 4 |
| oviduct opening state | one side opening is not visible | 1 |
|  | bilateral openings are not visible | 2 |
|  | barrel cavity, bilateral horn disappeared | 4 |
| endometrial thickness (late proliferation) | ≥7 mm | 1 |
|  | 4~6mm | 2 |
|  | ≤3 mm | 4 |
| menstrual status | menstrual volume less than 1/2 normal volume | 1 |
|  | guttata | 2 |
|  | amenorrhea | 4 |
| previous pregnancy history | spontaneous abortion once | 1 |
|  | recurrent abortion | 2 |
|  | infertility | 4 |
| history of curettage | induced abortion | 1 |
|  | curettage in early pregnancy | 2 |
|  | curettage in the second and third trimester | 4 |
| Note:mild：total score 0 ~ 8 points；moderate：total score: 9 ~ 18points；severe：total 19-28 points | | |

supplemental data
